# 

**Published:** 1856-10

**Authors:** 


Our readers will notice, in the present number, that our available space is mostly occupied by two articles; that by Dr. Reeves on Typhoid Fever, and that by Prof. Hamilton, on Fractures. Both of these, though long, will repay careful perusal and study. Prof. Hamilton's article will be continued through a series of numbers, and will, when complete, embrace the most succinct and valuable mass of facts relative to the comparative frequency of different fractures, and their results, ever yet published.

In the article of Dr. Reeves, the northern reader will find some things to surprise him, in the frequency of typhoid fever in western Virginia. We call attention, especially, to the evidence of contagion there adduced. It is only in rural districts that such evidence can be properly traced. The history given by Dr. Reeves is similar to that of the "Hamburg Epidemic," as described by Prof. Flint, some years since. The latter has been quoted in Europe as the most direct and thorough proof of the contagiousness of typhoid fever ever published.

So far as treatment is concerned, it will be noticed that the early treatment is more active than at the north, that less reliance is placed on quinine, beef and brandy, and more on diluents. Depletion, however, is deprecated, and it is probable that the difference in locality justifies, and renders necessary, this difference in treatment.

